# Age-related metabolic and neurodegenerative changes in SAMP8 mice

**DOI:** 10.18632/aging.204284

**Published:** 2022-09-16

**Authors:** Andrea Pačesová, Martina Holubová, Lucie Hrubá, Veronika Strnadová, Barbora Neprašová, Helena Pelantová, Marek Kuzma, Blanka Železná, Jaroslav Kuneš, Lenka Maletínská

**Affiliations:** 1 Institute of Organic Chemistry and Biochemistry of the Czech Academy of Sciences, Prague 166 10, Czech Republic; 2 Institute of Microbiology of the Czech Academy of Sciences, Prague 142 00, Czech Republic; 3 Institute of Physiology of the Czech Academy of Sciences, Prague 142 00, Czech Republic

**Keywords:** Alzheimer’s disease, senescence accelerated mouse prone 8, neuroinflammation, tau pathology, insulin resistance

## Abstract

The most important risk factor for the development of sporadic Alzheimer’s disease (AD) is ageing. Senescence accelerated mouse prone 8 (SAMP8) is a model of sporadic AD, with senescence accelerated resistant mouse (SAMR1) as a control. In this study, we aimed to determine the onset of senescence-induced neurodegeneration and the related potential therapeutic window using behavioral experiments, immunohistochemistry and western blotting in SAMP8 and SAMR1 mice at 3, 6 and 9 months of age. The Y-maze revealed significantly impaired working spatial memory of SAMP8 mice from the 6^th^ month. With ageing, increasing plasma concentrations of proinflammatory cytokines in SAMP8 mice were detected as well as significantly increased astrocytosis in the cortex and microgliosis in the brainstem. Moreover, from the 3^rd^ month, SAMP8 mice displayed a decreased number of neurons and neurogenesis in the hippocampus. From the 6^th^ month, increased pathological phosphorylation of tau protein at Thr231 and Ser214 was observed in the hippocampi of SAMP8 mice. In conclusion, changes specific for neurodegenerative processes were observed between the 3^rd^ and 6^th^ month of age in SAMP8 mice; thus, potential neuroprotective interventions could be applied between these ages.

## INTRODUCTION

Neurological disorders, especially various types of dementia where Alzheimer’s disease (AD) accounts for 60% of all cases, are increasing in prevalence. The main reason is the ageing population, since ageing is the main risk factor for AD development [1]. Although the majority of patients suffer from sporadic/late onset forms of AD, research is frequently conducted on transgenic mouse models carrying mutations connected to familiar/early onset forms of AD, such as a Swedish mutation of amyloid precursor protein (APP), mutation of presenilin 1 (PS1), or mutated microtubule associated protein tau (MAPT) [2].

To date, no effective treatment is available to alleviate symptoms of AD, such as extracellular deposits of β-amyloid plaques (Aβ) and hyperphosphorylated tau protein, as two main hallmarks of AD, and reduced synaptic plasticity and neurogenesis [3]. Currently, the interconnection of inflammation, insulin resistance and neurodegeneration has been extensively examined [4]. The increased activation of microglia and astrocytes producing proinflammatory cytokines, e.g., interleukin 6 (IL-6) or tumor necrosis factor α (TNFα), was observed in the brains of AD patients and in AD rodent models [5–7]. Proinflammatory cytokines potentiate insulin resistance, which could further increase tau hyperphosphorylation [8]. Inflammation is present not only in the brain but also in the periphery. Chronic inflammation is often connected to obesity, where cytokines are produced by adipose tissue [9]. However, increased levels of proinflammatory cytokines were also observed with ageing; the term “inflamm-aging” was introduced by Fransceschi et al. [10].

One of the models mimicking sporadic AD is senescence accelerated mouse-prone 8 (SAMP8) mice with senescence accelerated mouse-resistant 1 (SAMR1) mice as controls, which were selected from the AKR/J strain and bred into inbred lines by the Takeda laboratory [11, 12]. SAMP8 mice are considered as a model for the study of therapeutic interventions for sporadic AD because they were reported to develop typical AD pathologies (mentioned above) [13]. The proper beginning of treatment with potentially neuroprotective drugs in SAMP8 mice is rather crucial, since the treatment has to be performed for weeks or months [14]. Moreover, it is necessary to take into account that the median survival time of SAMP8 mice is nearly half of that of SAMR1 controls [15]. Many studies focused on particular aspects of ageing and neurodegeneration were performed in SAMP8 mice in comparison to their SAMR1 controls, such as behavioral changes connected to ageing [16–18], different metabolic or neuropathological changes [19–23]. However, discrepancies are observed in the onset of particular pathologies related to neurodegeneration, as well as in metabolic profiles. For example, Hansen et al. reported that despite expectations, 10-month-old SAMP8 mice showed memory deficits but no significant increase in Aβ and tau hyperphosphorylation compared to both SAMR1 and 4-month-old SAMP8 controls [23]. On the other hand, Canudas et al. revealed significantly increased tau phosphorylation in several brain areas at the age of 5 months in SAMP8 mice [20]. Besides, slight hyperglycemia and increased levels of insulin were found with ageing in SAMP8 mice by Cuesta et al. [19], whereas Yan et al. observed decreasing insulin levels with ageing of SAMP8 mice [24].

A more detailed age profile of SAMP8 mice and their SAMR1 controls is therefore needed for assignment of behavior, memory, anatomical, and biochemical parameters related to AD manifestation markers. Thus, our present study is focused on the complex characterization of behavior, metabolic profile, including urine NMR metabolomics employed for the first time, and neuropathological changes in SAMP8 mice in comparison to their SAMR1 controls at several age points that are connected to the progression of AD, especially with respect to inflammation and insulin resistance in the periphery, as well as central insulin resistance, neuroinflammation, tau hyperphosphorylation, and potential decrease in synaptogenesis and neurogenesis. In this way, we aimed to find the most suitable age window for testing potential neuroprotective compounds.

## RESULTS

### Behavioral experiments

Behavioral experiments were performed to monitor several aspects of the behavior of SAMP8 mice compared to their SAMR1 controls at the age of 3, 6 and 9 months; activity of mice was examined in the open field, anxiety was evaluated in the open field and elevated plus maze, or spatial working memory was assessed in the Y-maze.

### Increased activity and decreased anxiety of SAMP8 mice

**Figure 1:**
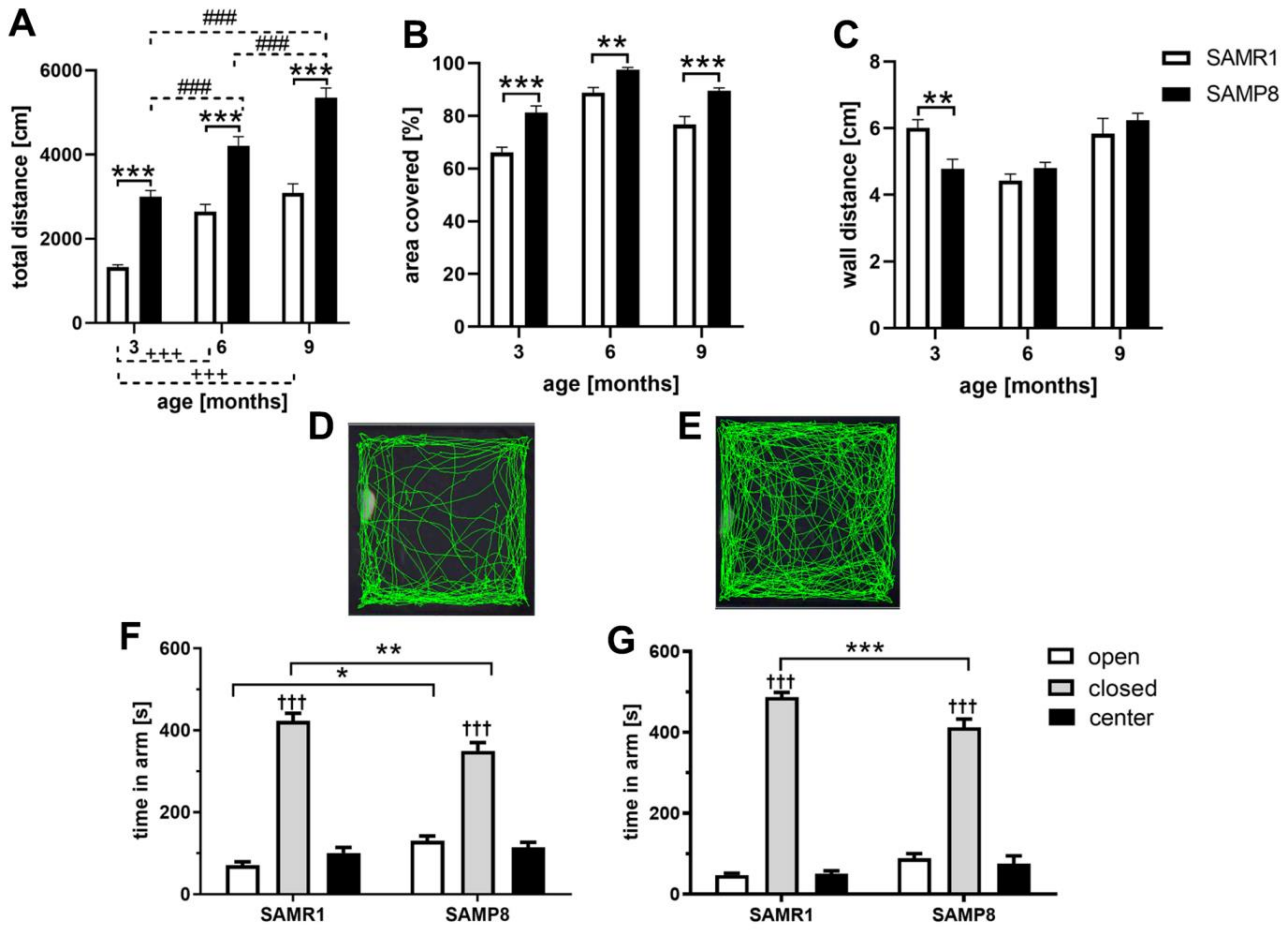
Increased activity measured by open field (**A**–**E**) and decreased anxiety measured by elevated plus maze (**F**, **G**) of SAMP8 mice. Mice spent 10 minutes in open field where the: (**A**) total distance (**B**) covered area (**C**) wall distance were measured. The representative tracks of 6 months old (**D**) SAMR1 mice (**E**) SAMP8 mice. Mice spent 10 minutes in elevated plus maze: (**F**) 6 months old mice (**G**) 9 months old mice. Data are mean ± SEM, analyzed by 2-way ANOVA, repeated measures, with Bonferroni post test. Significance is *P < 0.05, **P < 0.01 and ***P < 0.001. *SAMP8 mice compared to SAMR1, + age-dependent changes in SAMR1, # age-dependent changes in SAMP8, and ^†^ compared closed arm to open arm. n = 12 (3 months old), 10 (6 months old) or 5 (9 months old) mice per group.

During the 10 minutes that mice spent in the open field, SAMP8 mice from the age of 3 months ran a significantly longer total distance than their SAMR1 controls, as shown in Figure 1A, and covered a significantly larger area of the ground (Figure 1B); the representative tracks of the SAMR1 and SAMP8 mice at the age of 6 months are shown in Figure 1D, 1E, respectively. Wall distance was significantly decreased only in 3-month-old SAMP8 mice, which indicates higher anxiety compared to SAMR1 mice (Figure 1C). To further examine anxiety-like behavior, the elevated plus maze was performed at the age of 6 and 9 months. Compared to the open arms, both SAMR1 and SAMP8 mice at both ages spent more time in the closed arms (Figure 1F, 1G). However, at the age of 6 months, SAMP8 mice spent significantly more time in the open arms than SAMR1 controls; at the age of 9 months, only a tendency toward increased time was observed. At both ages, SAMP8 mice spent less time in the closed arms than SAMR1 controls.

### Impaired spatial working memory of the SAMP8 mice in Y maze

**Figure 2:**
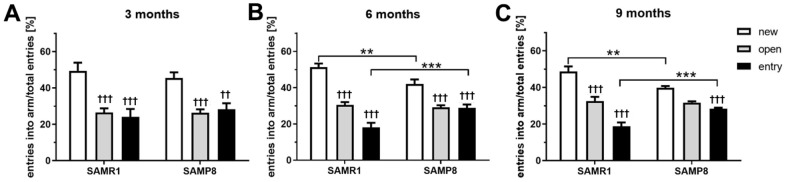
Impaired spatial working memory of SAMP8 mice in Y maze (**A**) 3 months old mice (**B**) 6 months old mice (**C**) 9 months old mice. Mice spent 5 minutes in the entry and open arm, then 5 minutes in free access to new, open and entry arms. Data are mean ± SEM, analyzed by 2-way ANOVA, repeated measures, with Bonferroni post test. Significance is **P < 0.01 and ***P < 0.001. * SAMP8 compared to SAMR1 mice, and ^†^ open and entry arm compared to the new arm. n = 12 (3 months old), 10 (6 months old) or 5 (9 months old) mice per group.

To evaluate spatial working memory, the Y maze test was performed at the age of 3, 6, and 9 months (Figure 2A–2C, respectively). From the age of 6 months, SAMP8 mice spent significantly less time in the new arm of the maze, as well as increased time in the known entry arm, indicating decreased spatial working memory compared to SAMR1 mice.

### Lower body weight in SAMP8 mice

**Figure 3:**
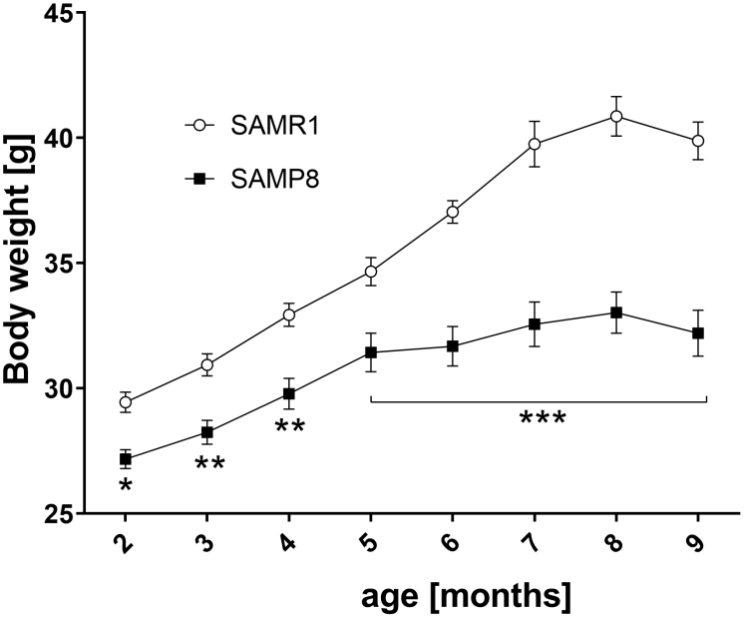
**Lower body weight SAMP8 mice from 2 months of age.** Body weight was measured every month. Data are mean ± SEM, analyzed by 2-way ANOVA with Bonferroni post test. n=14-15 (2-3 months), 10 (4-6 months), 5 (7-9 months).

As shown in Figure 3, from the 2^nd^ month of age, the SAMP8 mice were significantly leaner than their SAMR1 controls; this phenomenon was enhanced during the ageing.

In concordance with lower body weight, a lower weight of white adipose tissue (WAT, subcutaneous and epididymal) was observed at the ages of 6 and 9 months in SAMP8 mice than in SAMR1 controls (Table 1) and a significantly decreased level of the adipokine leptin was detected at the age of 9 months. On the other hand, no differences were observed in the plasma levels of triacylglycerols (TAGs), or free fatty acids (FFAs). The concentration of plasma cholesterol was significantly lower in 6- and 9-month-old SAMP8 mice than in SAMR1 mice. There were no significant differences in the concentration of blood glucose between SAMR1 and SAMP8 mice. The level of plasma insulin did not differ between SAMP8 mice and their controls.

**Table 1:** Metabolic parameters of SAMR1 and SAMP8 mice.

		**WAT**	**Leptin**	**FFAs**	**TAGs**	**Cholesterol**	**Glucose**	**Insulin**
		**[g]**	**[ng/ml]**	**[mmol/l]**	**[mmol/l]**	**[mmol/l]**	**[mmol/l]**	**[ng/ml]**
**3 M**	**SAMR1**	0.27 ± 0.06	0.19 ± 0.04	0.27 ± 0.05	0.52 ± 0.08	2.57 ± 0.10	9.3 ± 0.6	0.54 ± 0.18
	**SAMP8**	0.39 ± 0.07	0.48 ± 0.19	0.21 ± 0.03	0.74 ± 0.07	2.01 ± 0.04	11.4 ± 1.7	0.37 ± 0.11
**6 M**	**SAMR1**	0.78 ± 0.10	0.35 ± 0.17	0.37 ± 0.03	0.65 ± 0.05	2.79 ± 0.30	8.9 ± 1.8	0.05 ± 0.02
	**SAMP8**	0.42 ± 0.17	0.94 ± 0.69	0.51 ± 0.12	0.81 ± 0.12	1.76 ± 0.17 **	6.5 ± 1.1	0.10 ± 0.02
**9 M**	**SAMR1**	1.47 ± 0.17	2.94 ± 0.68	0.45 ± 0.04	0.54 ± 0.07	3.09 ± 0.08	10.0 ± 0.7	0.19 ± 0.09
	**SAMP8**	0.46 ± 0.13 ***	0.85 ± 0.11 ***	0.34 ± 0.04	0.48 ± 0.14	1.86 ± 0.27 **	8.8 ± 0.6	0.16 ± 0.02

Data are mean ± SEM analyzed by 2-way ANOVA, Bonferroni post-hoc test. *P < 0.05, **P < 0.01 and ***P < 0.001 (*SAMP8 compared to SAMR1 at the same age), n = 4-5 mice per group.

### Increased rectal temperature and increased cytokine levels in SAMP8 mice

**Figure 4:**
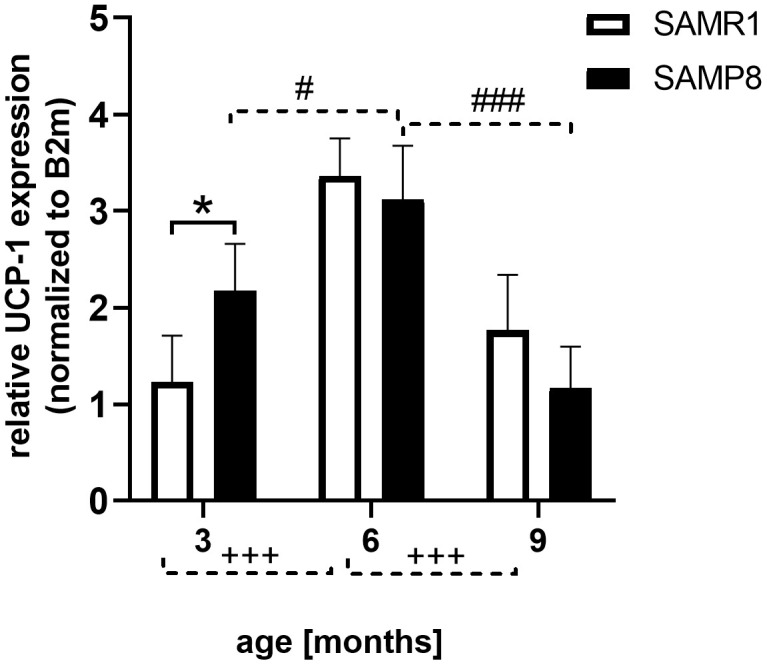
**Increased mRNA expression of UCP-1 in brown adipose tissue of 3 months old SAMP8 mice.** Data are mean ± SEM, analyzed by 2-way ANOVA with Bonferroni post test. Significance is *P < 0.05, and ***P < 0.001; *SAMP8 mice compared to SAMR1, +age-dependent changes in SAMR1, and # age-dependent changes in SAMP8. n = 4-5 mice per group.

SAMP8 mice had significantly increased rectal temperature compared to age-matched SAMR1 mice (shown in Table 2). In brown adipose tissue (BAT), mRNA expression of uncoupling protein 1 (UCP-1), a marker of increased thermogenesis, was significantly higher in SAMP8 mice than in SAMR1 mice only at the age of 3 months, increasing significantly between the 3^rd^ and 6^th^ months of age in both SAMP8 mice and their respective controls, as shown in Figure 4. Between the 6^th^ and 9^th^ months, decreased expression of UCP-1 was observed in SAMP8 mice.

**Table 2:** Rectal temperature, and level of CRP and cytokines in blood plasma of SAMR1 and SAMP8 mice.

		**Rectal temperature**	**CRP**	**TNFα**	**IL-6**
		**[° C]**	**[μg/ml]**	**[pg/ml]**	**[pg/ml]**
**3 M**	**SAMR1**	37.0 ± 0.2	4.50 ± 0.30	2.51 ± 0.52	2.16 ± 0.70
	**SAMP8**	37.9 ± 0.2*	3.20 ± 0.69*	2.48 ± 0.92	4.09 ± 1.29
**6 M**	**SAMR1**	37.0 ± 0.1	4.16 ± 0.86	3.09 ± 1.56	4.03 ± 0.39
	**SAMP8**	38.3 ± 0.3***	6.02 ± 0.44**	4.64 ± 1.01	20.64 ± 12.04
**9 M**	**SAMR1**	36.4 ± 0.2	4.30 ± 0.76	4.15 ± 0.69	18.21 ± 4.39
	**SAMP8**	37.4 ± 0.3*	4.61 ± 0.40	6.66 ± 3.20	36.22 ± 8.10*

Data are mean ± SEM analyzed by 2-way ANOVA, Bonferroni post-hoc test. *P < 0.05, **P < 0.01 and ***P < 0.001 (* SAMP8 compared to SAMR1 at the same age), n = 5 mice per group.

At the age of 3 and 6 months, level of c-reactive protein (CRP) was significantly increased in SAMP8 mice compared to their controls. Concentration of proinflammatory cytokines TNFα and IL-6 tended to increase in SAMP8 mice from the 6^th^ month. Significantly increased concentration of IL-6 was observed in blood plasma of 9-month-old SAMP8 mice compared to SAMR1 (as shown in Table 2).

### Worsened neuroinflammation with ageing in the brains of SAMP8 mice

**Figure 5:**
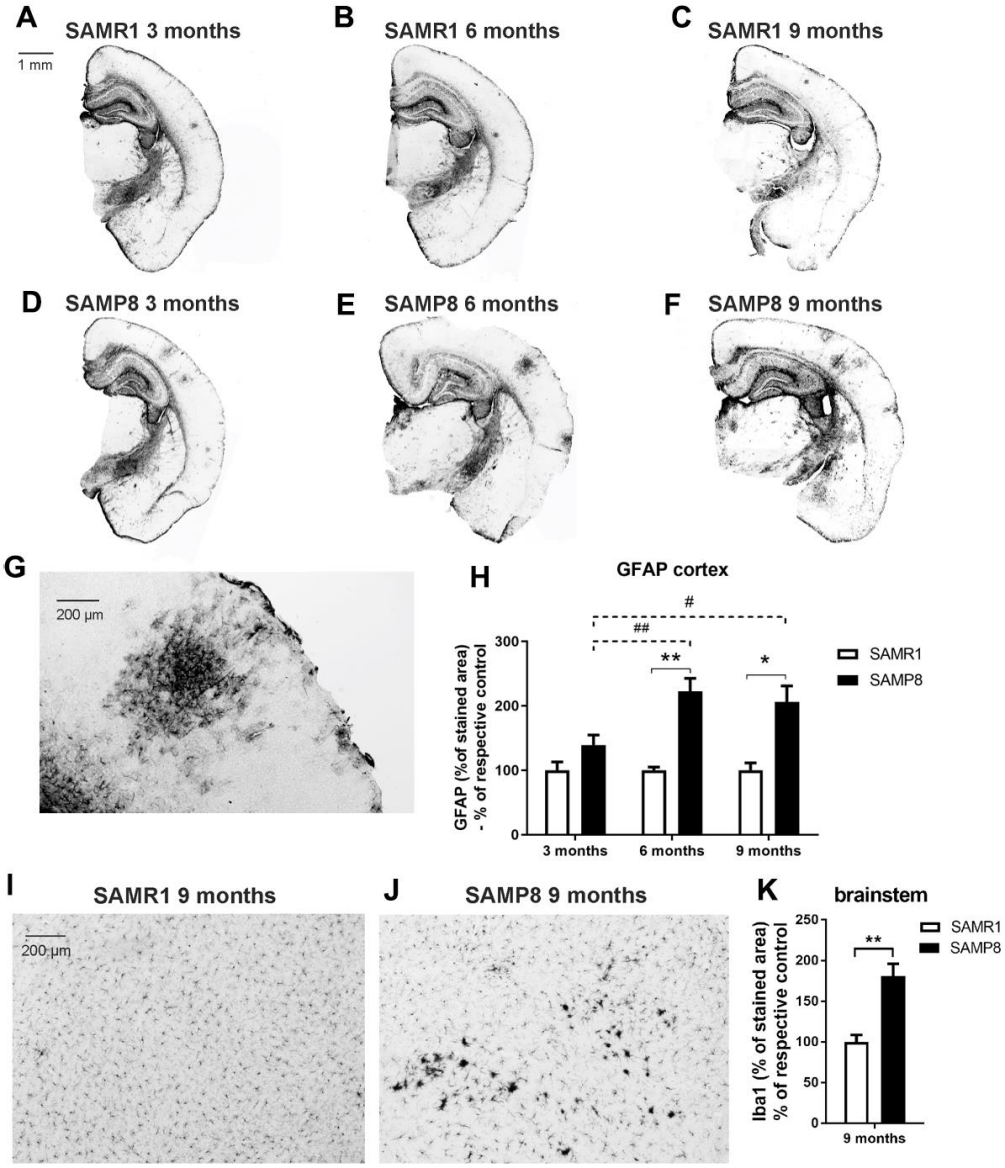
Increased neuroinflammation in the brains of SAMP8 mice: (**A**–**G**) marker of reactive astrocytes GFAP in the cortex, and (**H**) its quantification, and (**I**, **J**) marker of microglia Iba1 in the brainstem and (**K**) its quantification. Data are mean ± SEM, analyzed by 2-way ANOVA with Bonferroni post test (GFAP) or t-test (Iba1). Significance is *P < 0.05 and **P < 0.01. * SAMP8 compared to SAMR1, + age-dependent changes in SAMR1, and # age-dependent changes in SAMP8. n = 4-5 mice per group, 8-10 sections per brain.

Six- and 9-month-old SAMP8 mice showed significantly increased glial fibrillary acidic protein (GFAP) reactivity in the cortex compared to the age-matched controls; a similar trend was also observed in the 3-month-old mice. Figure 5A–5H illustrates the visible clusters of GFAP-stained reactive astrocytes and the quantification of the immunohistochemical staining (IHC).

Staining with ionized calcium-binding adapter molecule 1 (Iba1), a marker expressed by both resting and activated microglia and macrophages, revealed clusters of activated microglia in the brainstems of 9-month-old SAMP8 mice in contrast to the age-matched controls which did not develop any microglial activation (Figure 5I, 5J); the percentage of the area stained with Iba1 was significantly increased in the SAMP8 mice compared to the SAMR1 controls (Figure 5K). However, in the hippocampus and cortex, there were no visible clusters in SAMR1 or SAMP8 mice of any age, and no significant difference in the intensity of Iba1 staining was found between the strains (Supplementary Figure 1A–1C).

### Decreased number of neurons and neurogenesis in the brains of SAMP8 mice

**Figure 6:**
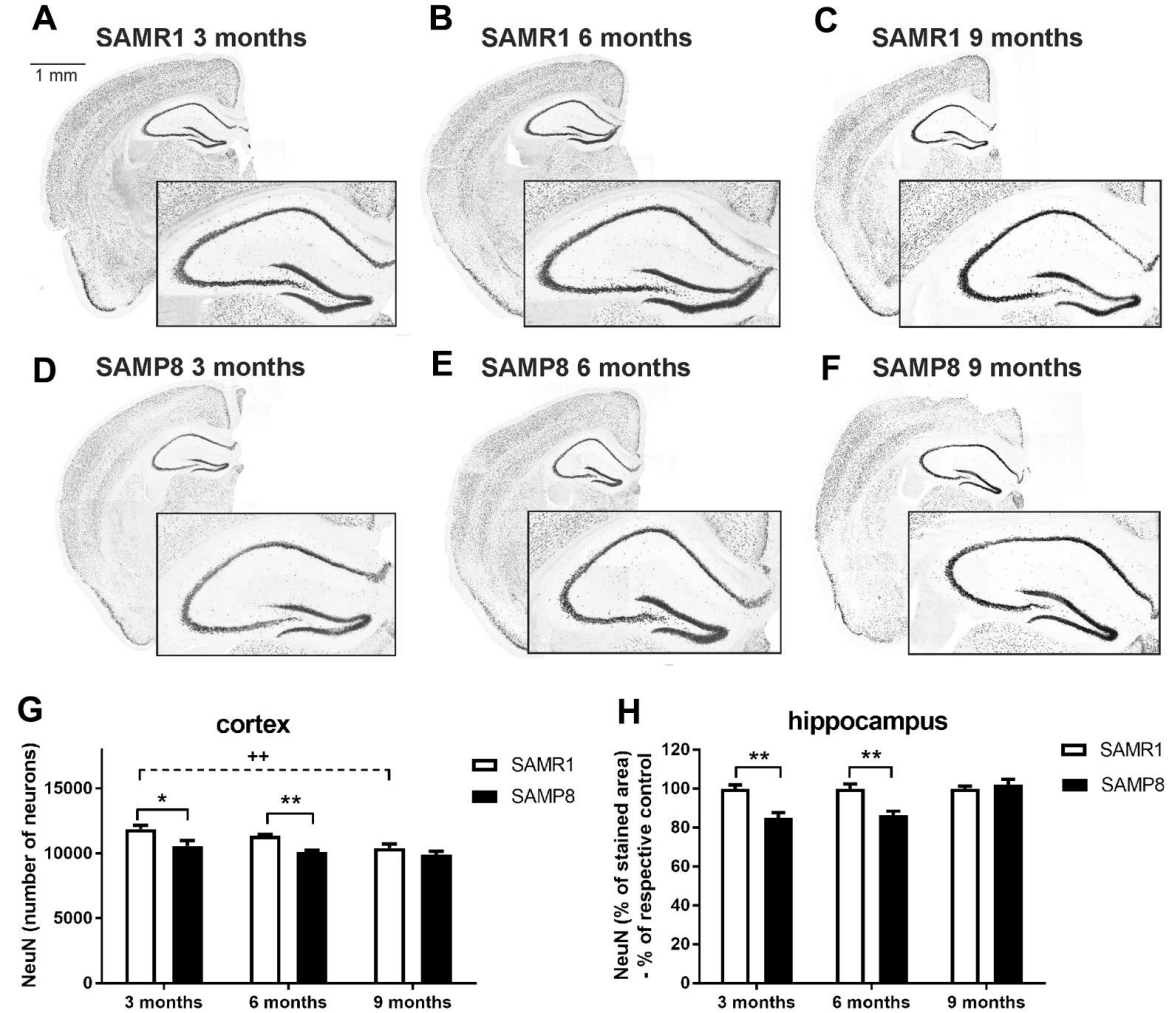
Decreased total number of neurons in the cortex and hippocampus of SAMP8 immunohistochemically stained for NeuN: representative photomicrographs of the brains of SAMR1 (**A**–**C**) and SAMP8 (**D**–**F**) mice. Black-framed inserts in right down corners show a magnified area of hippocampus, and (**G**, **H**) the quantification. Total number of stained particles was counted in cortex, percentage of the stained area expressed as a % of a respective control group was used in hippocampus. Data are mean ± SEM, analyzed by 2-way ANOVA with Bonferroni post test. Significance is *P < 0.05 and **P < 0.01. * SAMP8 compared to SAMR1,+ age-dependent changes in SAMR1, and # age-dependent changes in SAMP8. n = 4-5 mice per group, 8-10 sections per brain.

Three- and 6-month-old SAMP8 mice showed significantly decreased number of neurons (marked by NeuN, Figure 6) in both the cortex and hippocampus compared to the age-matched controls (SAMR1 mice). At the age of 9 months, there was no significant difference between strains; however, the number of neurons was reduced in the SAMR1 controls compared to those 3 months old.

**Figure 7:**
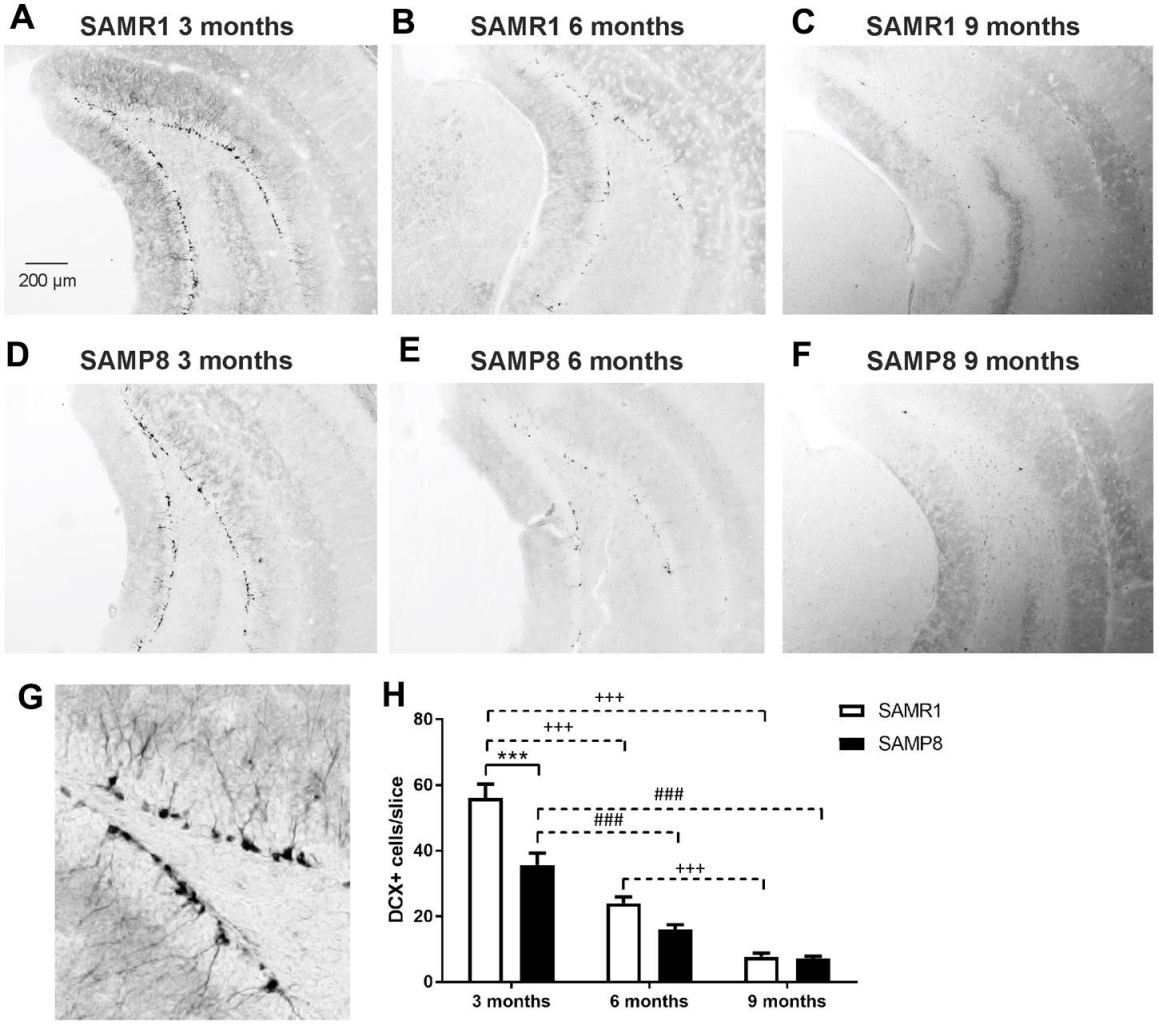
Decreased neurogenesis in the hippocampi of SAMP8 mice immunohistochemically stained for doublecortin (DCX): representative photomicrographs of the brains of SAMR1 (**A**–**C**) and SAMP8 (**D**–**F**) mice, (**G**) a detail of DCX-stained neurons in the subgranular zone of the hippocampus, and (**H**) the quantification. Data are mean ± SEM, analyzed by 2-way ANOVA with Bonferroni post test. Significance is ***P < 0.001. * SAMP8 compared to SAMR1, + age-dependent changes in SAMR1, and # age-dependent changes in SAMP8. n = 4-5 mice per group, 8-10 sections per brain.

Similarly, adult neurogenesis, measured as the number of doublecortin (DCX)-positive cells, was significantly reduced in 3-month-old SAMP8 mice compared to the age-matched control strain. The same trend was observed in 6-month-old mice, but the difference did not reach significance. The number of newly generated neurons dropped sharply with increasing age in both strains, and at the age of 9 months, there was no apparent difference between strains (Figure 7).

### Decreased synaptic plasticity in the hippocampi of SAMP8 mice

**Figure 8:**
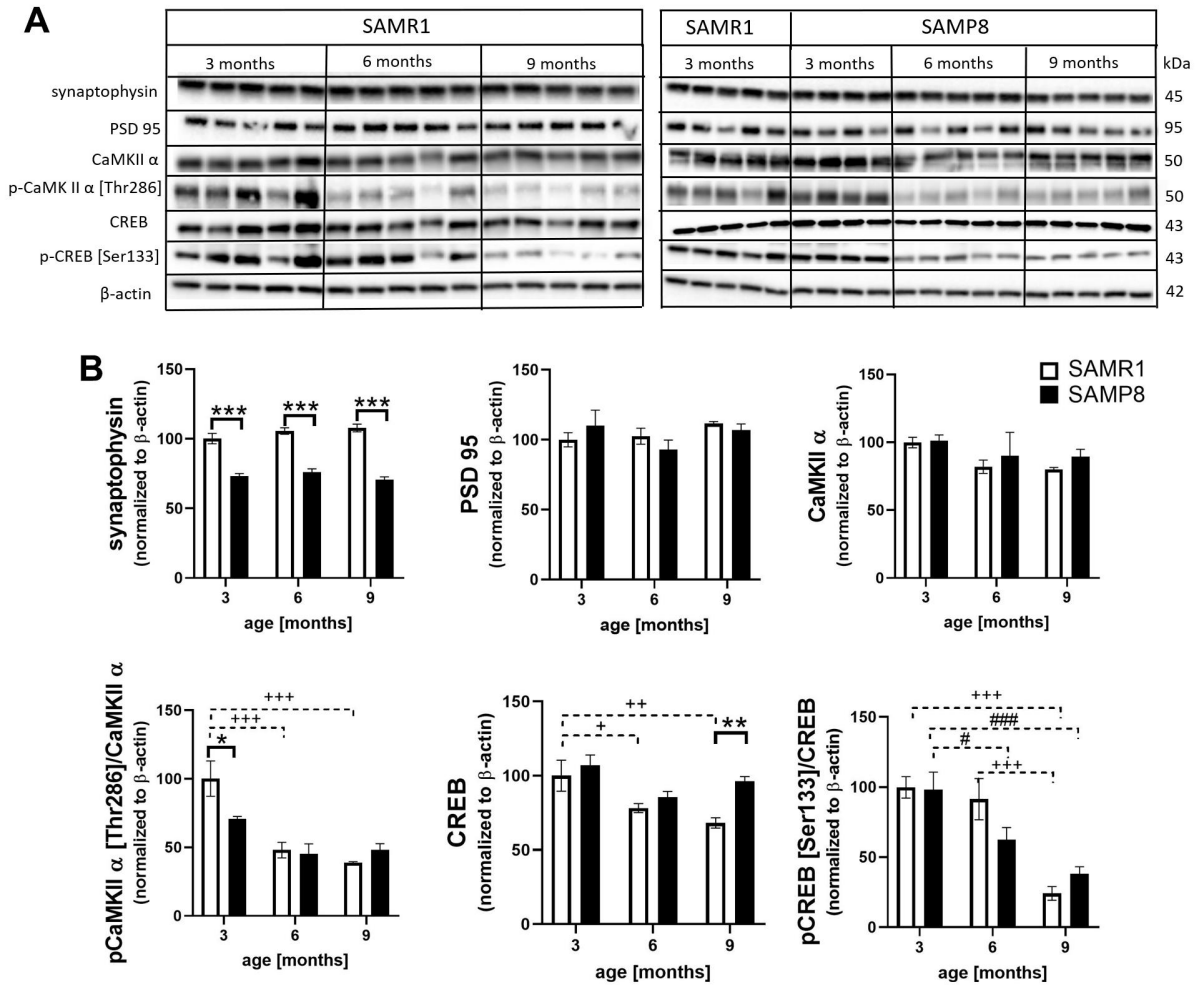
Decreased markers of synaptic plasticity in the hippocampi of SAMP8 (**A**) western blots and (**B**) their quantification. Data are mean ± SEM, analyzed by 2-way ANOVA with Bonferroni post test. Significance is *P <0.05, **P < 0.01 and ***P < 0.001. * SAMP8 compared to SAMR1, + age-dependent changes in SAMR1, and # age-dependent changes in SAMP8. n = 4-5 mice per group.

At all examined ages, significantly decreased levels of the presynaptic marker synaptophysin were observed in SAMP8 mice compared to SAMR1 controls (Figure 8). The level of postsynaptic density protein 95 (PSD95) did not differ at any age between SAMP8 and SAMR1 mice. The level of the most abundant postsynaptic density protein, calcium/calmodulin-dependent protein kinase II α (CaMKIIα), did not differ between strains and with ageing; however, its activating phosphorylation at Thr286 was significantly lower in SAMP8 mice than in SAMR1 mice at 3 months of age. CaMKIIα is a kinase of c-AMP-response element binding (CREB), and analogously, significantly decreased activating phosphorylation at Ser133 was observed in the hippocampi of SAMP8 mice with ageing.

### Impaired activation of the insulin signaling cascade in the hippocampi of SAMP8 mice

**Figure 9:**
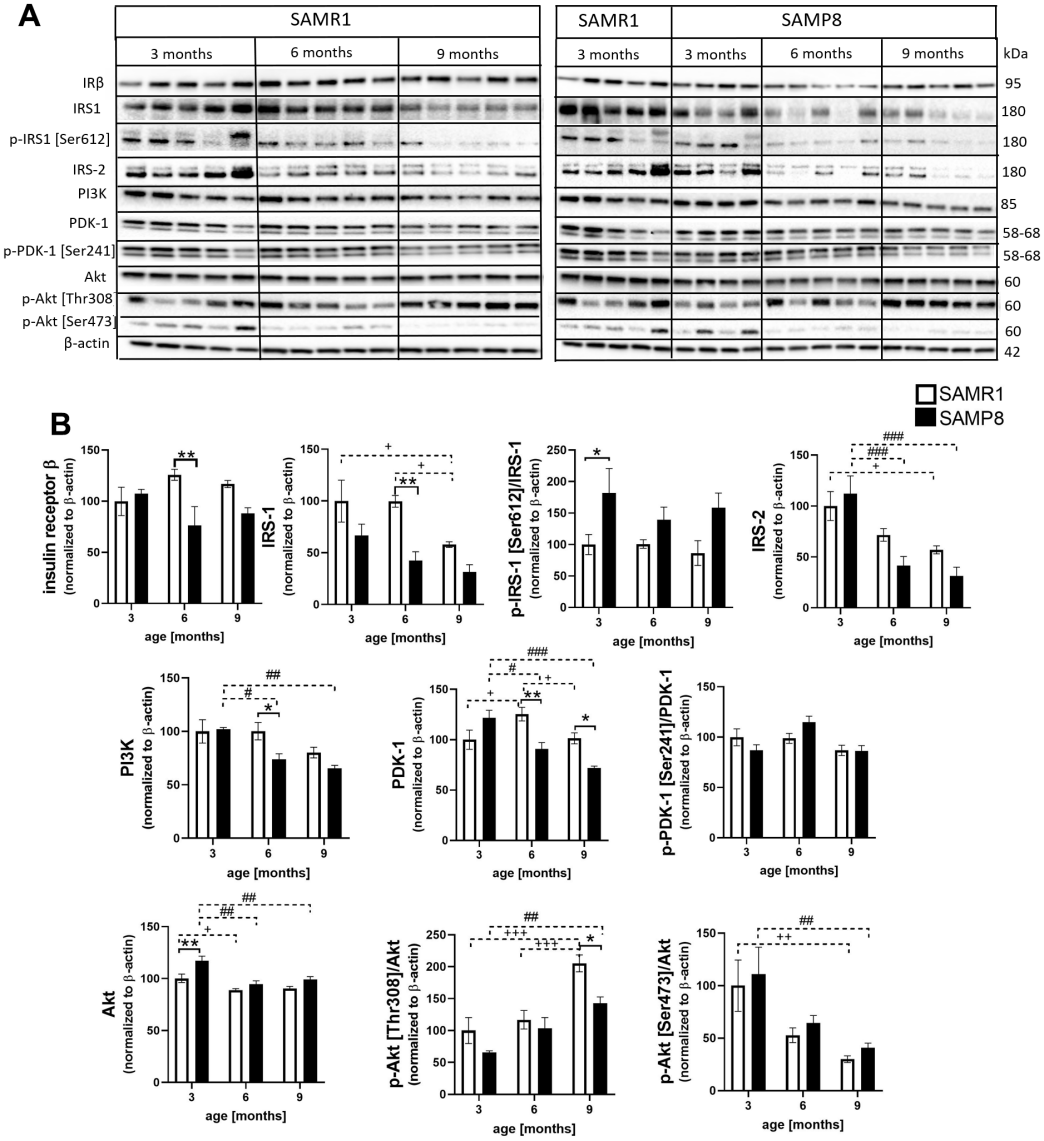
Impaired activation of the insulin signaling cascade in the hippocampi of SAMP8 mice (**A**) western blots and (**B**) their quantification. Data are mean ± SEM, analyzed by 2-way ANOVA with Bonferroni post test. Significance is *P < 0.05, **P < 0.01 and ***P < 0.001; * SAMP8 compared to SAMR1, + age-dependent changes in SAMR1, and # age-dependent changes in SAMP8. n = 4-5 mice per group.

In the hippocampi of SAMP8 mice, insulin resistance was observed (Figure 9). First, SAMP8 mice showed decreased hippocampal insulin receptor β at the age of 6 months compared to SAMR1 controls. Insulin receptor substrate 1 (IRS-1) decreased significantly with age in SAMR1 mice; a nonsignificant decrease was observed at 3-month-old SAMP8 mice in comparison with SAMR1 mice. Significantly increased phosphorylation of IRS-1 at Ser612 was observed in 3-month-old SAMP8 mice. Decreasing levels of IRS-2 was observed with ageing in SAMP8 mice; however, they did not differ from those in age-matched SAMR1 mice. Additionally, kinases implicated in the insulin signaling cascade, such as phosphoinositide 3-kinases (PI3K) and phosphoinositide-dependent kinase-1 (PDK-1), were reduced in SAMP8 mice compared to SAMR1 controls. Phosphorylation of Akt at Thr308 increased in SAMR1 mice with ageing; on the other hand, in SAMP8 mice, phosphorylation was less pronounced and was significantly lower at 9 months of age in SAMP8 mice than in SAMR1 controls. Phosphorylation of Akt at Ser473 decreased with ageing in both SAMR1 and SAMP8 mice; no differences were observed between SAMR1 and SAMP8 mice.

### Decreased activity of tau phosphatase PP2A in the hippocampi of SAMP8 mice

**Figure 10:**
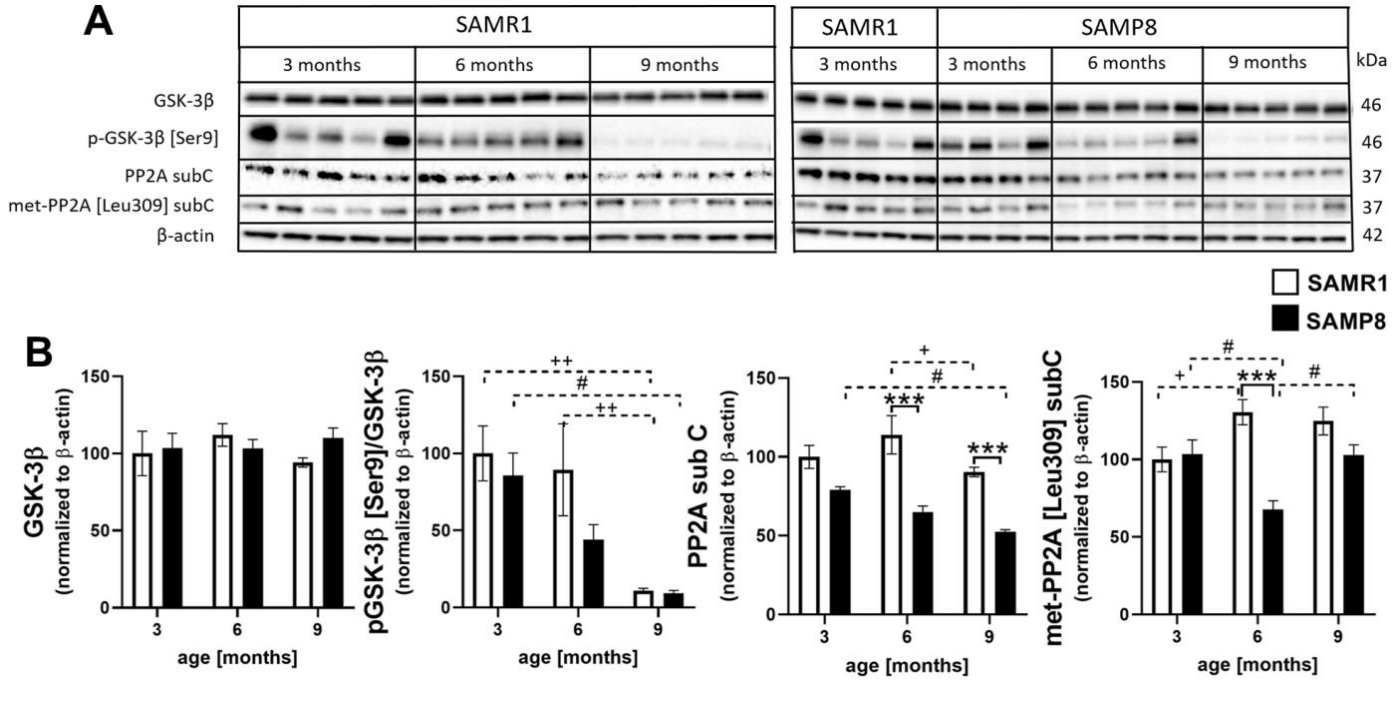
Increased activation of tau kinase GSK-3β and decreased activation of protein phosphatase 2A sub C in the hippocampi of SAMP8 (**A**) western blots and (**B**) their quantification. Data are mean ± SEM, analyzed by 2-way ANOVA with Bonferroni post test. Significance is *P < 0.05, **P < 0.01 and ***P < 0.001; * SAMP8 compared to SAMR1, + age-dependent changes in SAMR1, and # age-dependent changes in SAMP8. n = 4-5 mice per group.

The level of total glycogen synthase kinase 3β (GSK-3β), one of the main tau kinases that is also implicated in the insulin signaling cascade, did not differ during ageing or between the SAMR1 and SAMP8 mice at any age (Figure 10). However, phosphorylation at the inhibitory epitope Ser9 significantly decreased during the ageing of both strains. Total level of main tau phosphatase called protein phosphatase 2A (PP2A) was significantly lower in SAMP8 mice compared to SAMR1, at the age of 6 and 9 months; in concordance, the level of PP2A methylated at Leu9, which is the marker of its activation, also significantly decreased at 6-month-old SAMP8 mice and nonsignificantly at the age of 9 months.

### Hyperphosphorylation of tau protein at different epitopes in the hippocampi of SAMP8 mice

**Figure 11:**
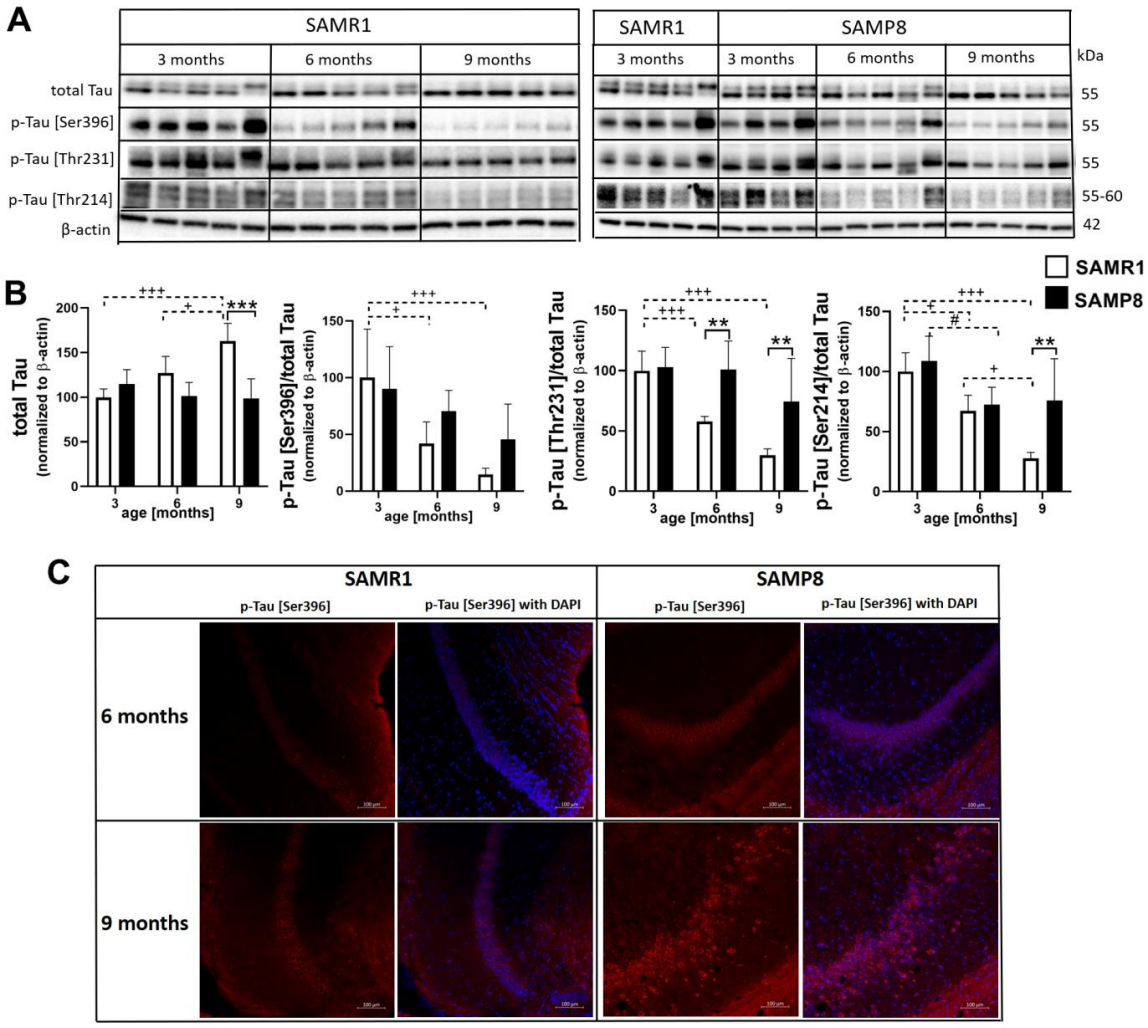
Hyperphosphorylation of tau protein in the hippocampi of SAMP8 mice (**A**) western blots, (**B**) their quantification, and (**C**) immunofluorescence of p-tau [Ser396] at CA1 area of the hippocampus. Data are mean ± SEM, analyzed by 2-way ANOVA with Bonferroni post test. Significance is *P < 0.05, **P < 0.01 and ***P < 0.001; * SAMP8 compared to SAMR1, + age-dependent changes in SAMR1, and # age-dependent changes in SAMP8. n = 4-5 mice per group.

In SAMR1 mice, increased levels of total tau protein with ageing were observed in the hippocampi (Figure 11). SAMP8 mice had increased phosphorylation of tau protein at different epitopes, such as Ser396, Thr231 and Ser214 in comparison with age-matched SAMR1 controls at the age of 6 or 9 months. Increased tau phosphorylation at Ser396 was confirmed by immunofluorescence; a more intense fluorescence signal was observed in the CA1 area of the hippocampus of SAMP8 mice than in SAMR1 mice at the ages of 6 and 9 months.

In addition to tau hyperphosphorylation, AD is characterized by the accumulation of Aβ plaques. However, in the brains of SAMP8 mice, no Aβ plaques were detected using IHC (Supplementary Figure 2).

### Increased peripheral insulin resistance in the skeletal muscle of SAMP8 mice

**Figure 12:**
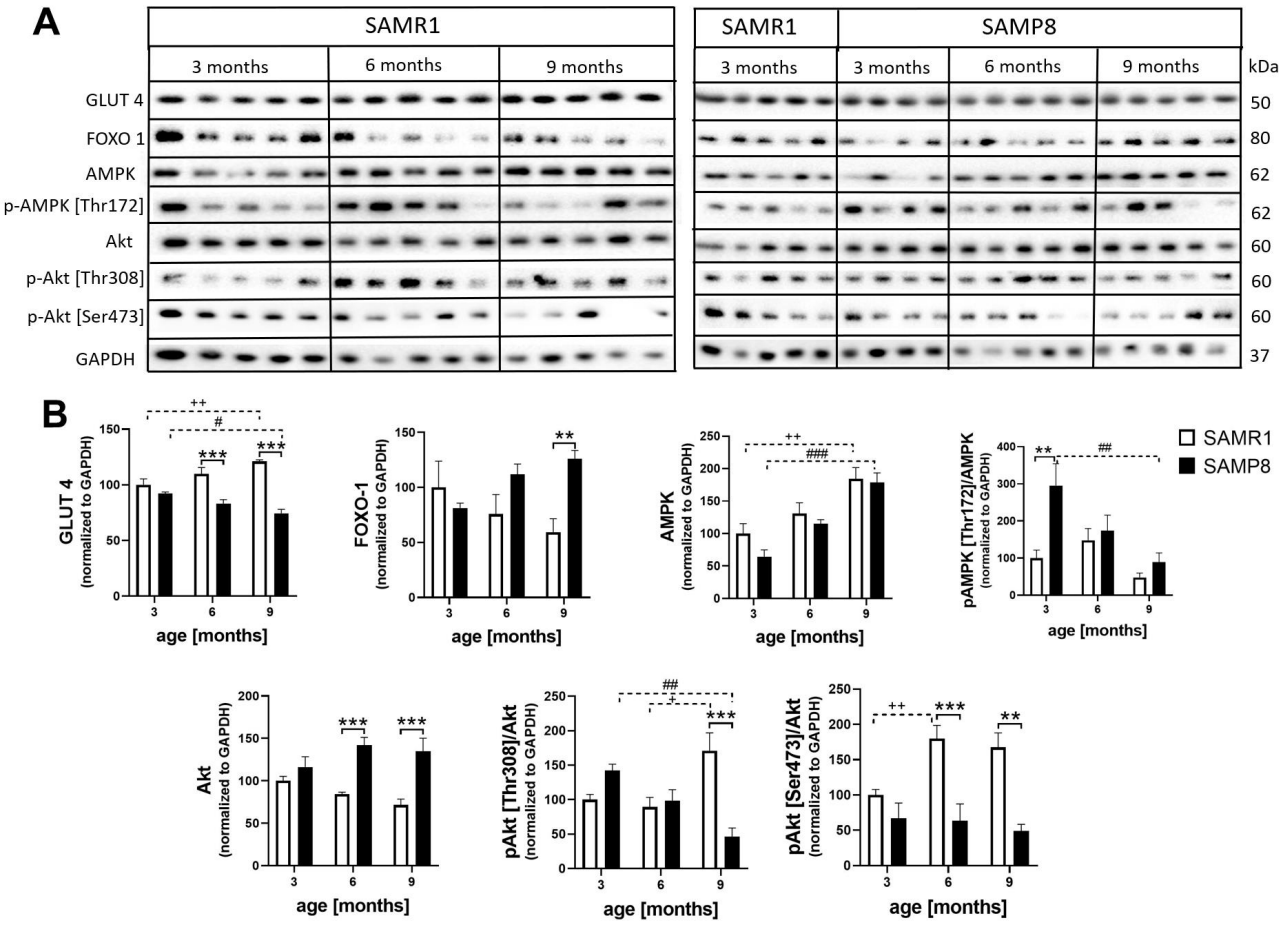
Increased insulin resistance in skeletal muscle of SAMP8 (**A**) western blots and (**B**) their quantification. Data are mean ± SEM, analyzed by 2-way ANOVA with Bonferroni post test. Significance is *P <0.05, **P < 0.01 and ***P < 0.001; * SAMP8 compared to SAMR1, + age-dependent changes in SAMR1, and # age-dependent changes in SAMP8. n = 4-5 mice per group.

In skeletal muscle (Figure 12), SAMP8 mice showed significantly decreased glucose transporter 4 (GLUT 4) from the 6^th^ month and increased levels of forkhead box protein O1 (FoxO1) at the age of 9 months compared to controls. The level of total AMP-activated protein kinase (AMPK) did not differ between SAMR1 and SAMP8 mice; however, the level increased with ageing in both strains. SAMP8 mice had significantly increased levels of AMPK phosphorylated at Thr172 at the 3^rd^ month. A significantly increased level of total Akt was observed in SAMP8 mice from the 6^th^ month; on the other hand, significantly decreased Akt activation manifested by significantly decreased phosphorylation at Thr308 and Ser473 was observed in 9-month-old SAMP8 mice and at Ser473 in 6-month-old SAMP8 mice.

### NMR metabolomics revealed significant differences in urine composition between SAMP8 and SAMR1 mice at the age of 3 months

The differences between urinary metabolic profiles of both strains were evaluated by both multivariate and univariate approaches. PCA did not detect any outliers in the SAMP8 or SAMR1 groups. Then, a PLS-DA model was built; leave-one-out cross-validation showed satisfactory values (as shown in Supplementary Figure 3).

The highest VIP score of the PLS-DA model identified bins, corresponding to the signals of metabolites responsible for group separation. The difference between SAMP8 mice and their SAMR1 controls at 3 months of age is caused mainly by the combination of increased levels of trimethylamine, dimethylamine, citrate, and N-carbamoyl-β-alanine together with decreased concentrations of creatine, methanol, n-hexanoylglycine, butyrylglycine and vinylacetylglycine. As the multivariate approach is based on the simultaneous evaluation of all metabolite levels and the model is characterized as the unique combination of their changes, univariate statistics were used to describe the significance of changes in individual metabolite concentrations.

Within a set of sixty signals quantified in urine spectra, twenty metabolites were evaluated as significantly changed in SAMP8 mice compared to SAMR1 controls and are summarized in Table 3.

**Table 3:** Significantly changed metabolites in 3-month-old SAMP8 mice.

**Metabolite**	**NMR signal used for the quantitation**	**SAMP8 vs. SAMR1 ∆ %**
1-Methylnicotinamide	8.90 (m)	29.4 ^**^
3-Indoxylsulfate	7.71 (m)	52.2 ^*^
Acetate	1.92 (s)	-59.1 ^*^
Butyrylglycine	1.61 (m)	-23.9 ^**^
Carnitine	3.23 (s)	50.6 ^**^
Creatine (phosphate)	3.93 (s)	-53.9 ^**^
Creatinine	4.06 (s)	23.7 ^**^
Dimethylamine	2.72 (s)	76.5 ^***^
Glycine	3.55 (s)	71.7 ^**^
n-Hexanoylglycine	0.88 (t)	-17.9 ^*^
Methanol	3.37 (s)	-51.5 ^*^
Methylamine	2.61 (s)	32.3 ^*^
N-Carbamoyl β-alanine	3.31 (m)	58.3 ^**^
Niacinamide	8.72 (m)	-15.0 ^*^
Nicotinamide N-oxide	8.75 (m)	36.4 ^*^
Orotate	6.20 (s)	49.2 ^**^
Putrescine	1.85 (m)	-38.1 ^***^
Tartrate	4.34 (s)	137.3 ^*^
Trimethylamine	2.88 (s)	155.9 ^**^
Vinylacetylglycine	1.88 (dd)	-36.1 ^**^
Unknown triplet	3.17 (t)	65.0 ^***^

The results are expressed as the percentage change of concentration in SAMP8 vs. SAMR1 analyzed by Wilcoxon-Mann-Whitney test. *P < 0.05, **P < 0.01 and ***P < 0.001. Signal multiplicity is marked as follows: (s)-singlet, (t)-triplet, (dd)-doublet od doublets, (m)-multiplet.

## DISCUSSION

For late-onset AD, which accounts for 90% of AD cases, the most important risk factor is ageing [1]. Many symptoms connected to ageing and neurodegeneration have been described, such as memory impairment, changes in behavior, increased neuroinflammation, Aβ plaque load, tau hyperphosphorylation, or decreased neurogenesis and synaptogenesis; however, the mechanism of their progression is not fully understood [3]. For the present study, SAMP8 mice with accelerated ageing were employed to examine the ageing processes leading to the development of neurodegeneration.

One of the first symptoms of dementia is cognitive decline [25]. Thus, a set of behavioral experiments focused on changes connected to the progression of neurodegenerative diseases, especially basic exploratory activity in the open field and anxiety-like behavior [26], as well as spatial working memory, such as the Y-maze [27], were performed. In our study, SAMP8 mice from the age of 3 months showed increased mobility compared to SAMR1 controls, which is in concordance with the observations of Sawano et al. [16] or Tsumagari et al. [28]; the mobility of SAMP8 mice compared to SAMR1 significantly continued to increase with ageing. Moreover, similar to a study with 4-month-old SAMP8 mice performed by Yanai and Endo [29], we observed significantly decreased wall distance in 3-month-old SAMP8 compared with SAMR1 mice, which pointed to increased anxiety-like behavior; however, no such differences were observed at the ages of 6 and 9 months. In contrast, SAMP8 mice showed an increased covered area in the open field from the 3^rd^ month of age compared to SAMR1 mice, similar to Sawano et al. [16]. To better explore anxiety-like behavior of SAMP8 mice, the elevated plus maze, which is based on the natural aversion of mice for open and elevated areas on the one hand and natural spontaneous exploratory behavior on the other hand, was performed. SAMP8 mice at the age of 6 and 9 months showed less anxiety-like behavior than SAMR1 control, which was manifested by less time spent in the closed arms of the elevated plus maze and by a significantly prolonged time spent in the open arms at the age of 6 months. Similar trend in tendency to spend more time in the open arms of elevated plus maze was observed in SAMP8 mice compared to their SAMR1 control at the age of 5 months by Fujiwara et al. [18]. Spatial working memory of SAMP8 mice observed in the Y-maze was significantly worsened at the age of 6 and 9 months which is consistent with results reported by del Valle at these ages [17], by Gong et al. in SAMP8 mice at the age of 6 months [30], or by Lin et al. at the age of 10 months [31]. From the age of 6 months, SAMP8 mice had significant memory deficits.

One of the first observed signs of SAMP8 mice was significantly lowered body weight from the 2^nd^ month compared to SAMR1 controls which corresponded to a decreased WAT from the 6^th^ month, consequently decreased levels of leptin in plasma in 9-month-old SAMP8 mice, and decreased levels of cholesterol; no difference in FFAs or TAGs concentration was observed in SAMP8 mice compared to SAMR1 controls. Inflammation is often associated with obesity (reviewed by Kacířová et al. [9]) when increased levels of proinflammatory cytokines, such as TNFα or IL-6, are produced by adipose tissue. Concentrations of IL-6 significantly and TNFα nonsignificantly increased in the blood plasma of SAMP8 mice with ageing. This increase in the plasma in our study could be connected with increased rectal temperature of SAMP8 mice at the ages of 6 and 9 months, since TNFα and IL-6 were defined as possible inducers of fever [32, 33]. In 3-month-old SAMP8 mice, where no differences in cytokines were observed between SAMP8 and SAMR1, the increased rectal temperature could be connected to increased expression of UCP-1 in BAT, which can increase thermogenesis of the mice [34], or to increased levels of CRP.

In the brain, neuroinflammation is manifested by elevated levels of GFAP, a marker of astrogliosis, or Iba1, a marker of microgliosis; neuroinflammation could contribute to memory impairments, and further negatively influence adult neurogenesis [21]. Although increased activation of microglia was observed only in the brainstem of 9-month-old SAMP8 mice, confirming the observation of Yagi et al. [35], increased levels of GFAP were observed in the brain cortex of SAMP8 mice; a nonsignificant increase was observed in mice at the age of 3 months and further escalated at the ages of 6 and 9 months, in accordance with Sawano et al., who observed increased levels of GFAP form the 3^rd^ month [16]. Memory impairment is further connected to neuronal loss, especially in the hippocampus, which corresponds to the severity of AD [36]. In agreement with that observation, neuronal loss manifested by decreased NeuN was found in SAMP8 mice from the 3^rd^ month in the brain cortex and the hippocampus, which further progressed at the age of 6 months. In contrast to Li et al. [22], who observed increased neuronal loss in SAMP8 mice at the age of 8 months, in the present study, no differences were observed between SAMP8 and SAMR1 mice at that age. As a compensatory mechanism for the diminishing number of neurons, increased neurogenesis manifested by an increased number of DCX-positive cells was observed in SAMP8 mice by Gang et al. [37]; neurogenesis decreased with ageing between the 5^th^ and 10^th^ months of age. However, SAMP8 mice in our study displayed a significantly decreased number of DCX-positive cells compared to SAMR1 already at the age of three months, which further progressed with ageing.

Another important player contributing to impaired spatial memory and cognitive decline is decreased synaptic plasticity, mainly in the hippocampus as the center of learning and memory formation, which can be potentiated by neuroinflammation. In concordance, we observed significantly reduced amounts of the synaptic marker synaptophysin in the hippocampi of SAMP8 mice compared with SAMR1 mice at all examined ages, as was observed by del Valle in 6- and 9-month-old SAMP8 mice [17]. On the other hand, no such difference was observed in postsynaptic density proteins, such as PSD95 or CaMKIIα. However, for proper CaMKII function, autophosphorylation at Thr286 is crucial [38], and from the 3^rd^ month of age, decreased phosphorylation of CaMKIIα at this epitope was observed in SAMP8 mice compared with SAMR1 controls. CREB, which is important for long-term potentiation and memory formation, could be phosphorylated by CaMKIIα [39, 40], and its decreased activation was manifested by diminished phosphorylation at Ser133 with ageing in SAMP8 mouse hippocampi.

In addition to synaptic plasticity, neuroinflammation can negatively influence the insulin signaling cascade, and impaired activation of the insulin signaling cascade can further cause hyperphosphorylation of tau protein, one of the hallmarks of AD [41], through increased kinase activity of GSK-3β toward tau protein caused by decreased phosphorylation at inhibitory epitope Ser9. Decreased central insulin signaling is also connected to impaired spatial memory or decreased synaptic plasticity [42, 43]. In this study, we first observed nonsignificantly decreased levels of insulin receptor β in the hippocampi of SAMP8 mice compared to SAMR1 controls, with a significant decrease at the age of 6 months. A similar trend was described by Yan et al., who explored the insulin receptor in the brains of 3- and 9-month-old SAMP8 mice [24]. The level of IRS-2, whose deletion is known to increase tau phosphorylation and lead to improper brain development [44], was reduced with ageing in the hippocampi of SAMP8 mice. The same trend was observed in the level of IRS-1, whose activation was impaired by increased phosphorylation at Ser612, the phosphorylation connected to insulin resistance [45]. Other kinases implicated in the insulin signaling cascade, such as PI3K, PDK1 and Akt, were also reduced with ageing in SAMP8 mice compared to SAMR1 controls. Decreased phosphorylation of Akt at Thr308 was manifested in the hippocampi of SAMP8 mice at the age of 9 months, and age-dependent decreased phosphorylation at Ser473 was observed in both SAMP8 and SAMR1 mice. Akt is a kinase that inhibits GSK-3β kinase activity toward tau protein by phosphorylation at Ser9 [46]. Decreased phosphorylation of inhibitory Ser9 at GSK-3β was observed at the age of 6 months in the hippocampi of SAMP8 mice, and increased tau phosphorylation was detected at Ser396, Thr231 and Ser214, similar to a study with 5-month-old SAMP8 mice by Canudas et al. [20]. The tau protein is under the control of many kinases and phosphatases, and an increase in phosphorylation of tau protein can be caused by decreased activation/methylation of the main tau phosphatase PP2A [47]. In the hippocampi of SAMP8 mice, decreased levels of PP2A were found, with attenuated methylation at Leu309 at the age of 6 months; our data are in concordance with Yang et al. [48] and Zhen et al. [49] who observed decreased phosphatase activity in SAMP8 mice at the age of 6 and 9 months, respectively.

Although plasma concentrations of insulin and glucose did not differ between SAMP8 and SAMR1 mice, decreased levels of the glucose transporter GLUT4 and attenuated activation of Akt were observed in skeletal muscle of 6- and 9-month-old SAMP8 mice, pointing to peripheral insulin resistance, which is usually observed in patients with T2DM [50]. Moreover, an increase in FOXO-1 levels with ageing is connected to decreased levels of GLUT4 [51], inhibition of the insulin signaling cascade and reduced muscle mass [52]. Significantly increased activation of AMPK was observed in 3-month-old but not older SAMP8 mice; such activation was observed in cachectic mice and can be connected to increased concentrations of IL-6 [53]. Our data confirm the observation of Liu et al. [54], who defined SAMP8 mice as a suitable model of increased inflammation and subsequent insulin resistance.

To obtain a deeper insight into the biochemical background of metabolic changes in SAMP8 mice, NMR-based metabolomic analysis of urine samples was applied. To date, several mass spectrometry-based metabolomic studies of blood or cerebrospinal fluid (CSF) sample models have been published for the SAMP8 model, often following the effects of various factors (food supplements, drugs, treatment, etc.) on the development or improvement of AD metabolomics [55–58]. NMR-based characterization of the SAMP8 model was performed by Jiang et al. [59] on serum samples of 12-month-old SAMP8 and SAMR1 mice. Authors identified serum inosine as the most important metabolite for strain separation and described more significant metabolic deviation in females than males.

The present metabolomic study, which takes advantage of the complex composition and noninvasive collection of urine samples, revealed significant differences between substantially younger male SAMP8 and SAMR1 mice at the age of 3 months. Significantly attenuated urinary excretion of putrescine in the SAMP8 strain was observed. Putrescine is a polyamine that is known to possess anti-inflammatory activity via the inhibition of inflammatory cytokine synthesis in macrophages [60].

Recently, AD has been associated with changes in the gut microbiome composition [61]. In our study, increased methylamine, dimethylamine, and trimethylamine concentrations were detected. Methylamines are the gut microbiota degradation products of dietary choline [62], which is – as a precursor of acetylcholine - an important metabolite for brain development. Trimethylamine N-oxide, the oxidation product of trimethylamine, was elevated in the CSF of AD patients [63]. 3-Indoxylsulfate is another gut microbial metabolite that is elevated in the urine of SAMP8 mice. This toxin displays proinflammatory effects by acting on glial cells in the brain, resulting in increased production of cytokines, such as IL-6 or TNFα [64] and, thus, could contribute to neurodegeneration. To summarize, the reported changes in urinary microbial metabolites pointed to the different microbial activities in SAMP8 mice and were connected to increased inflammation compared to SAMR1 controls.

It is well known that nicotinamide adenine dinucleotide/nicotinamide adenine dinucleotide hydrate (NAD+/NADH) homeostasis plays a pivotal role in AD; its imbalance in the hippocampus and cortex results in elevated production of reactive oxygen species and increased neuroinflammation [65, 66]. Dalmasso et al. observed a significant decrease in nicotinamide in plasma of AD patients even approximately one year prior to AD onset [67]. Our study showed a reduced amount of nicotinamide and an increase in its metabolites in the urine of 3-month-old SAMP8 mice. We speculate that this indicates the close relationship between NAD+ metabolism and AD onset. Elevated levels of 1-methylnicotinamide were demonstrated as a potential biomarker of peroxisome proliferation [68] and linked to inflammation, obesity and metabolic syndrome [69]; its increased urinary concentration was observed in rodent models of obesity and was also suggested as a biomarker for diabetes [70–72].

Carnitine is crucial for the metabolism of FFAs [73]. Among other things, it neutralizes the brain damage induced by oxidative stress. Its rise in 3-month-old SAMP8 mice is consistent with the observation of Wang et al., who detected higher carnitine levels in the SAMP8 model at 6 months of age [55]. Moreover, our study also revealed increased glycine levels and a reduction in several acylglycines, hexanoylglycine, n-butyrylglycine, and vinylacetylglycine, which are minor metabolites of fatty acids. Therefore, the presented changes may imply that mitochondrial dysfunction is connected with AD.

Creatine, which was detected at lower levels in SAMP8 urine, participates in energy metabolism and has a therapeutic effect in various diseases [74]. Moreover, its potential neuroprotective effect was shown using rat hippocampal neurons; carnitine prevents cell death when neurons were exposed to Aβ or glutamate [75]. Decreased creatine concentrations were observed in the hippocampi of AD patients [76].

## CONCLUSIONS

Altogether, our data indicate that SAMP8 mice, with accelerated age, from their young age, already between the 3^rd^ and 6^th^ months, exhibit early onset pathologies connected to neurodegenerative changes, such as peripheral and central inflammation together with insulin resistance in the periphery, as well as in the hippocampus, where increased tau phosphorylation, as one of the hallmarks of AD, was observed. All these pathologies are also manifested by early onset impairment of spatial memory and altered anxiety-like behavior of SAMP8 mice. Nevertheless, the onset of the pathologies, which are not as robust as in transgenic mouse models of AD, is dependent on the experimental conditions and can differ among the laboratories.

SAMP8 mice with accelerated ageing seem to be a suitable mouse model to study potentially neuroprotective compounds with treatment beginning at the age of 3 months, since the pathological hallmarks can be observed from the 3^rd^ month of age. The most significant changes were in the behavior of the SAMP8 mice in comparison to the SAMR1 controls, such as increased locomotor activity, decreased anxiety, and impaired spatial working memory. Moreover, metabolic alterations in SAMP8 mice manifested as changes in the urinary metabolic profile compared to SAMR1 controls. Furthermore, a decreased number of neurons, neurogenesis, synaptogenesis and increased neuroinflammation were observed, as shown in Table 4 where all these changes are summarized.

**Table 4:** Summary of measured parameters connected to neurodegeneration and metabolic profile in SAMP8 mice compared to SAMR1 controls.

	**Parameter**	**SAMP8 vs SAMR1**		
		**3 months**	**6 months**	**9 months**
Behavioral tests	Locomotor activity (open field)	↑↑↑	↑↑↑	↑↑↑
	Anxiety (elevated plus maze)	NM	↓↓	↓↓↓
	Spatial working memory (Y-maze)	-	↓↓	↓↓
Morphometry and Level of biochemical parameters in blood plasma	Body weight	↓↓	↓↓↓	↓↓↓
	WAT	-	-	↓↓↓
	leptin	-	-	↓↓↓
	insulin	-	-	-
	glucose	-	-	-
	rectal temperature	↑	↑↑↑	↑
	CRP	↑	↑↑	-
	IL-6	-	-	↑
	UCP-1	↑	-	-
IHC	Microgliosis (hipp)	-	-	-
	Microgliosis (brain stem)	-	-	↑↑
	Astrocytosis (cortex)	-	↑↑	↑
	NeuN (cortex)	↓	↓↓	-
	NeuN (hipp)	↓↓	↓↓	-
	DCX (hipp)	↓↓↓	-	-
	Aβ (hipp)	-	-	-
Synaptic plasticity in hipp (WB)	Synaptophysin	↓↓↓	↓↓↓	↓↓↓
	PSD95	-	-	-
Central insulin resistance in hipp (WB)	Insulin receptor β	-	↓↓	-
	p-IRS1 [Ser612]	↑	-	-
	PI3K	-	↓	-
	p-Akt [Thr308]	-	-	↓
tau kinase and Phosphatase in hipp (WB)	p-GSK-3β [Ser9]	-	-	-
	PP2A subC	-	↓↓↓	↓↓↓
tau protein in hipp (WB)	Total tau	-	-	↓↓↓
	p-Tau [Thr214]	-	↑↑	↑↑
	p-Tau [Thr231]	-	-	↑↑
Insulin resistance in skeletal muscle (WB)	GLUT4	-	↓↓↓	↓↓↓
	FOXO-1	-	-	↑↑
	p-Akt [Thr308]	-	-	↓↓↓
	p-Akt [Ser473]	-	↓↓↓	↓↓

Differences in SAMP8 mice compared to SAMR1 controls, analyzed by 2-way ANOVA with Bonferroni post test. ↑ higher/↓ lower in SAMP8 mice compared to SAMR1. Significance is ↑/↓ P < 0.05, ↑↑/↓↓ P < 0.01, and ↑↑↑/↓↓↓ P < 0.001. CRP – C-reactive protein, Hipp – hippocampus, IHC- immunohistochemistry, IL-6 interleukin 6, NM – not measured, TNFα – tumor necrosis factor α, WAT – white adipose tissue, WB – western blotting, - nonsignificant change.

## MATERIALS AND METHODS

### Experimental animals

In our study, male mice were chosen because of their common susceptibility to development of insulin resistance [77, 78]. Moreover, we tried to avoid the possible changes connected to estrous cycle in female mice. Fourteen male SAMP8 mice and 15 age-matched SAMR1 control mice aged 4 to 6 weeks from Envigo (Gannat, France) were housed in the Animal Facility of the Institute of Organic Chemistry and Biochemistry, Czech Academy of Sciences (CAS), Prague, Czech Republic, with a 12-hour light/dark cycle and temperature of 22 ± 2° C. Mice were housed 5 per cage with free access to water and Ssniff® R/M-H diet (Ssniff Spezialdiäten GmbH, Soest, Germany) containing 33%, 9% and 58% calories from proteins, fats and carbohydrates, respectively.

### Experimental design

Body weight and rectal temperature of SAMR1 and SAMP8 mice were measured once per month. To study the behavioral changes, mice at the age of 3, 6, and 9 months underwent a set of behavioral experiments (described below). Subsequently, fasted mice at the same ages (n = 5 mice per group) were perfused, and blood plasma, various peripheral tissues and brain were collected to study potential peripheral and central pathological changes connected to ageing (described below).

### Behavioral experiments

### Open field

Mice were separately placed in a squared box (50 cm x 50 cm) for 10 minutes. Their locomotor activity (total distance, covered area of the floor and wall distance) was measured using VideoMot software (TSE Systems, Bad Homburg, Germany) and analyzed by Noldus (Noldus, Wageningen, Netherlands). Wall distance was defined as a distance of an animal from the closest wall of the maze at the given moment, and its average value was determined by Noldus (Noldus, Wageningen, Netherlands).

### Elevated plus maze

Mice spent 10 minutes in the elevated plus maze, 50 cm above the ground which had two arms open and two arms closed. The time spent in particular arms or in the central part of the maze was detected using VideoMot software (TSE Systems, Bad Homburg, Germany) and analyzed by Noldus software (Noldus, Wageningen, Netherlands).

### Y-maze test

The Y-maze consisted of three identical arms (26 cm x 4 cm): entry, open and new arms. On the walls of the maze, clues were placed to help the mouse orient in the maze. In the first session lasting for 5 minutes, the mouse was placed through the entry arm of the maze to the Y-maze, where only entry and open arms were opened. Afterward, the mouse was closed in the entry arm for 3 minutes. In the second session, the mouse spent 5 minutes in the Y-maze, where all three arms were opened. The time spent in every arm and number of visits in each arm were measured using VideoMot (TSE Systems, Bad Homburg, Germany), and analyzed by Noldus software (Noldus, Wageningen, Netherlands).

### Dissections

The night before the dissections, mice were placed in individual metabolic cages (Tecniplast, Buguggiate, Italy) with access to water where urine was collected for NMR metabolomic characterization; after the addition of sodium azide the urine samples were stored at -80° C. Blood of overnight fasted mice was collected from the tail veins, and blood plasma was separated and stored at -80° C. The mice were deeply anesthetized with pentobarbital (120 mg/kg of body weight, Sigma–Aldrich, St. Louis, MO, USA) and transcardially perfused with ice-cold 0.01 M phosphate-buffered saline (PBS) pH 7.4 supplemented with heparin (10 U/ml, Zentiva, Prague, Czech Republic). White (subcutaneous and epididymal) and brown adipose tissue, liver and skeletal muscle (gastrocnemius) were dissected and weighed. The brains were maintained on ice to prevent tissue degradation. The right hemisphere of the brain used for IHC was placed in 4% paraformaldehyde dissolved in 0.1 M PBS pH 7.4 for 24 hours, and subsequently stored at 4° C in 30% sucrose in 0.1 M PBS pH 7.4 with 0.01% sodium azide. The hippocampus and cortex from the left hemisphere used for western blotting (WB) were dissected, placed into cold lysis buffer (62.5 mM Tris-HCl buffer pH 6.8, 1% deoxycholate, 1% Triton X-100, 50 mM NaF, 1 mM Na_3_VO_4_ and complete protease inhibitor (Roche Applied Science, Mannheim, Germany)) and stored at -80° C.

### Measurement of biochemical parameters

Glucose was measured using a Glucocard glucometer (Arkray, Tokyo, Japan). The blood plasma concentration of leptin was measured using an ELISA kit (Millipore, St. Charles, MI, USA), free fatty acids (FFAs) were analyzed using a colorimetric assay (Roche Mannheim, Germany), insulin was analyzed using RIA kits (Millipore, St. Charles, MI, USA), and triacylglycerols (TAGs) and cholesterol were analyzed using colorimetric assays (Erba Lachema, Brno, Czech Republic). Plasma levels of CRP, TNFα and IL-6 were determined using ELISA (Thermo Fisher Scientific, Waltham, MA, USA). Relative mRNA expression of UCP-1 was measured in BAT using reverse transcription PCR, as previously described by Pražienková et al. [79]. The expression was normalized to beta 2 microglobulin (b2M).

### Immunohistochemistry

Chromogenic IHC was described earlier by Holubová et al. [80]. In short, PBS-washed slices were incubated in citrate buffer pH 6 (Sigma–Aldrich, St. Louis, MO, USA) in a water bath at 90° C for 30 min to enhance antigen recognition. The sections were incubated in 0.6% H_2_O_2_ to quench endogenous peroxidase activity (30 min at RT), permeabilized in 0.2% Triton X-100 in TBS (TBS-T; 20 min at RT), blocked in 5% normal goat serum (NGS) solution in TBS-T, and incubated overnight at 4° C with primary antibodies (listed in Table 5). Subsequently, the slices were incubated for 90 min at RT with biotinylated goat anti-rabbit IgG (Vectastain ABC Kit, Vector Laboratories, Burlingame, CA, USA) and finally stained in 3,3’ -diaminobenzidine solution (Vector Laboratories, Burlingame, CA, USA). Stained sections were viewed and imaged under a BX53 microscope equipped with a DP74 camera (Olympus, Tokyo, Japan). For NeuN, Iba1 and GFAP staining, the complete section area was imaged to cover the whole area of interest. For DCX staining, only the subgranular zone of the hippocampus was imaged (8-10 sections per mouse). The percentage of the stained area or the stained cell counts were then analyzed using ImageJ software (NIH, Bethesda, MD, USA). The area of interest was selected manually according to the mouse brain atlas [81]. Where the percentage of the stained area was used, the results were expressed as a percentage of the respective SAMR1 control group to enable comparison of different staining series. Immunofluorescence was also described earlier by Holubová et al. [80]. The method is identical to chromogenic IHC, except for H_2_O_2_ preincubation. Sections were incubated in secondary donkey anti-rabbit AlexaFluor594 (Thermo Fisher Scientific, Waltham, MA, USA) for 90 min at room temperature.

**Table 5:** List of primary antibodies with their appropriate dilution used in IHC.

**Primary antibody**	**Dilution**	**Manufacturer**
Doublecortin rabbit pAb	1 : 600	Cell Signaling Technology, Danvers, MA, USA
GFAP rabbit pAb	1 : 200	Thermo Fisher Scientific, Waltham, MA, USA
Iba1 rabbit mAb	1 : 2 000	Wako, Osaka, Japan
NeuN rabbit pAb	1 : 250	Thermo Fisher Scientific, Waltham, MA, USA
p-Tau [Ser396]	1 : 1 000	Thermofisher, Rockford, IL, USA

### Western blotting

The WB method was described earlier by Holubová et al. [80]. Briefly, the protein concentration of homogenized tissue was measured using a Pierce BCA protein assay kit (Thermo Fisher Scientific, Inc., Waltham, MA, USA) and subsequently dissolved in Laemmli sample buffer to a final concentration of 1 μg/μl. Samples were resolved using Criterion 4-15% precast gels (Bio–Rad, Hercules, CA, USA), transferred onto nitrocellulose membranes (Bio–Rad, Hercules, CA USA) and blocked in 5% nonfat milk or BSA in TBS/Tween-20 buffer (20 mM Tris, 136 mM NaCl, 0.1% Tween-20, 50 mM NaF, and 5 mM Na_3_VO_4_). After overnight incubation at 4° C in primary antibody (the appropriate dilution is shown in Table 6), membranes were incubated for 1 h at room temperature in HRP-linked secondary antibody (Cell Signaling Technology, Beverly, MA, USA) and developed using ECL solution Luminata Classico/Crescendo Western HRP Substrates (Merck Millipore, Darmstadt, Germany). Chemiluminescence was visualized in a ChemiDoc™ System (Bio–Rad, Hercules, CA, USA) and quantified using Image Lab Software (Bio–Rad, Hercules, CA, USA). The exact protein level on each membrane was normalized to β-actin or glyceraldehyde 3-phosphate dehydrogenase (GAPDH) as an internal loading control.

**Table 6:** List of primary antibodies with their appropriate dilution used in WB.

**Primary antibody**	**Diluent**	**Dilution**	**Manufacturer**
β-actin mouse mAb	5% milk	1 : 10 000	Sigma-Aldrich, St. Louis, MO, USA
Akt rabbit mAb	5% BSA	1 : 1 000	Cell Signaling Technology, Danvers, MA, USA
p-Akt [Thr308] rabbit mAb	5% BSA	1 : 1 000	Cell Signaling Technology, Danvers, MA, USA
p-Akt [Ser473] rabbit mAb	5% BSA	1 : 1 000	Cell Signaling Technology, Danvers, MA, USA
AMPK rabbit mAb	5% BSA	1 : 1 000	Cell Signaling Technology, Danvers, MA, USA
p-AMPK [Thr172] rabbit mAb	5% BSA	1 : 1 000	Cell Signaling Technology, Danvers, MA, USA
FoxO-1 rabbit mAb	5% BSA	1 : 1 000	Cell Signaling Technology, Danvers, MA, USA
GAPDH mouse mAb	5% milk	1 : 1 000	Cell Signaling Technology, Danvers, MA, USA
Glut4 mouse mAb	5% BSA	1 : 1 000	Cell Signaling Technology, Danvers, MA, USA
GSK-3β rabbit mAb	5% BSA	1 : 1 000	Cell Signaling Technology, Danvers, MA, USA
p-GSK-3 [Ser9] rabbit mAb	5% BSA	1 : 1 000	Cell Signaling Technology, Danvers, MA, USA
Insulin receptor β rabbit mAb	5% BSA	1 : 1 000	Cell Signaling Technology, Danvers, MA, USA
IRS-1 rabbit mAb	5% BSA	1 : 1 000	Cell Signaling Technology, Danvers, MA, USA
p-IRS-1 [Ser612] rabbit mAb	5% BSA	1 : 1 000	Cell Signaling Technology, Danvers, MA, USA
IRS-2 rabbit pAb	5% BSA	1 : 1 000	Cell Signaling Technology, Danvers, MA, USA
PDK-1 rabbit pAb	5% BSA	1 : 1 000	Cell Signaling Technology, Danvers, MA, USA
p-PDK-1 [Ser241] rabbit mAb	5% BSA	1 : 1 000	Cell Signaling Technology, Danvers, MA, USA
PI3K p85 rabbit mAb	5% BSA	1 : 1 000	Cell Signaling Technology, Danvers, MA, USA
PP2A subC rabbit mAb	5% BSA	1 : 1 000	Cell Signaling Technology, Danvers, MA, USA
methyl-PP2A C [L309] mouse mAb	5% milk	1 : 1 000	Millipore, Temecula, CA, USA
PSD95 rabbit pAb	5% BSA	1 : 1 000	Cell Signaling Technology, Danvers, MA, USA
Synaptophysin rabbit pAb	5% milk	1 : 5 000	Santa Cruz Biotechnology, Inc., Dallas, TX, USA
Tau5 mouse mAb	5% milk	1 : 5 000	Invitrogen Corp., Frederick, MD, USA
p-Tau [Ser199] rabbit pAb	5% BSA	1 : 1 000	Thermofisher, Rockford, IL, USA
p-Tau [Ser214] rabbit pAb	5% BSA	1 : 1 000	Thermofisher, Rockford, IL, USA
p-Tau [Ser396] rabbit pAb	5% BSA	1 : 10 000	Thermofisher, Rockford, IL, USA

To compare SAMR1 and SAMP8 mice at all ages, samples of 3-month-old SAMR1 mice were applied to every gel and the level was assessed like a baseline (100%).

### NMR-based metabolomics of urine

Since only negligible amounts of urine were collected from 6- and 9-month-old SAMR1 and SAMP8 mice, urine metabolic profiles were evaluated only for mice at 3 months of age. Prior to NMR analysis, samples were thawed at room temperature and centrifuged at 18.620 rcf for 5 minutes. A 200 μl supernatant was mixed with 340 μl H_2_O and 60 μl phosphate buffer (1.5 M KH_2_PO_4_ in D_2_O containing 2 mM NaN_3_ and 0.1% trimethylsilyl propionic acid (TSP), pH 7.4) and transferred to a 5-mm NMR tube.

All NMR experiments were performed on a 600 MHz Bruker Avance III spectrometer (Bruker BioSpin, Rheinstetten, Germany) equipped with a 5-mm TCI cryogenic probe head at 300 K. To eliminate the background of naturally occurring mouse urinary proteins [82], NMR analysis was based on a Carr-Purcell-Meiboom-Gill (CPMG) experiment with water presaturation (Bruker pulse sequence cpmgpr1d), acquired with the following parameters: presaturation during relaxation delay=4 s; number of scans (NS)=128; number of data points =64 k; spectral width (SW)=20 ppm; echo time=0.3 ms; and loop for T2 filter=126. A short *J*-resolved experiment with presaturation (NS=2, SW=16 ppm, TD=16 k, number of increments=40, SW=78.125 Hz in the indirect dimension, and relaxation delay=2 s) was executed for all samples to solve the problem with the signal overlap. Additional HSQC and TOCSY experiments were performed on a pooled sample (evaporated, diluted with 540 μl D_2_O and 60 μl phosphate buffer) to support metabolite identification.

The acquired data were processed using Topspin 3.5 software (Bruker BioSpin, Rheinstetten, Germany). Line broadening of 0.3 Hz was applied on free induction decays (FIDs) prior to Fourier transformation. The spectra were automatically phased, baseline corrected and referenced to the signal of TSP (0.00 ppm); regions with water and urea resonance (4.50-5.15 and 5.65-6.00 ppm, respectively) were excluded before further analysis. Probabilistic quotient normalization (PQN) [83] was performed using a pooled 3-month-old SAMR1 group as the reference.

### Statistical analysis

The data are presented as the means ± SEMs. Using GraphPad Prism 7 Software (San Diego, CA, USA), statistical analysis was performed using unpaired t test (Welch’s correction), two-way ANOVA followed by Bonferroni’s multiple comparisons test, or two-way ANOVA, repeated measures followed by Bonferroni’s multiple comparisons test in case of behavioral experiments, where the same animals underwent the behavioral experiments at the age of 3, 6 and 9 months. P < 0.05 was considered to be statistically significant.

For NMR metabolomics, untargeted multivariate analysis, based on the analysis of equidistantly binned spectra (bin width = 0.01 ppm), was performed in Metaboanalyst 4.0 software [84]. Principal component analysis (PCA) on Pareto scaled data was used to monitor sample distribution and detect potential outliers. Statistical models were built using partial least squares-discriminant analysis (PLS-DA) and then validated by leave-one-out cross-validation and permutation tests. The results of the PLS-DA model were evaluated using variable importance in projection (VIP) scores, which identified important bins contributing the most to the group separation.

Univariate analysis was performed on all well-resolved signals. Due to the small number of samples, the nonparametric Wilcoxon-Mann–Whitney test was applied using MATLAB software (version 9.0 R2016a). Individual metabolites were identified using Chenomx software (Chenomx Inc., Edmonton, AB, Canada) and by the comparison of acquired proton and carbon data with the HMDB database or with previously published data.

## Supplementary Material

Supplementary Figures
