# Supplementary material for: Age-related metabolic and neurodegenerative changes in SAMP8 mice
Source: Aging (Albany NY). 2022 Sep 16;14(18):7300–27. doi: 10.18632/aging.204284 (PMC9550245; doi:10.18632/aging.204284)
Supplement: Supplementary Figures [file aging-14-204284-s001.pdf]

SUPPLEMENTARY FIGURES

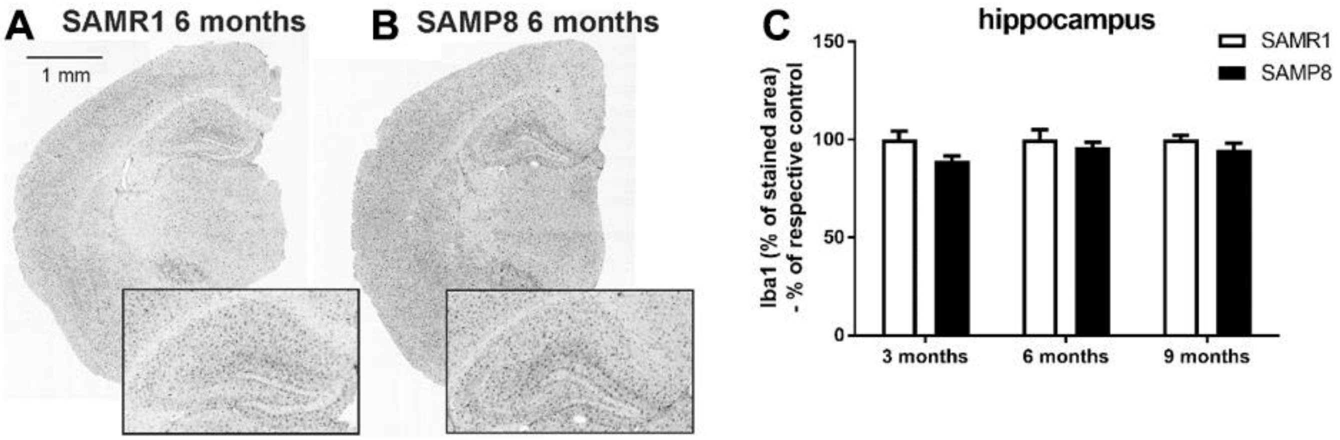

Supplementary Figure 1. No significant changes in the hippocampal microgliosis immunohistochemically stained with Iba1: (**A**, **B**) representative photomicrographs of the brains of 6-months-old mice, and (**C**) the quantification. Black-framed inserts in right down corners show a magnified area of hippocampus. Percentage of the stained area expressed as a % of a respective control group was used. Data are mean ± SEM, analyzed by t-test. No significant difference was found. n = 4-5 mice per group, 8-10 sections per brain.

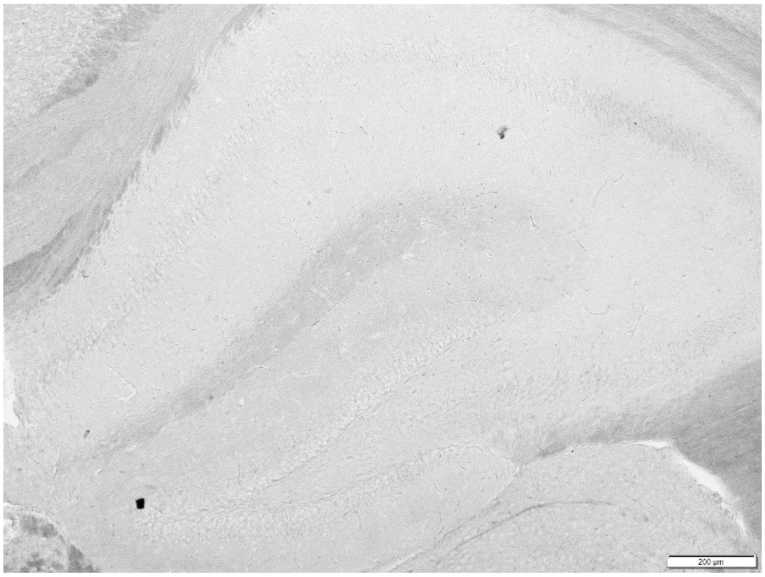

Supplementary Figure 2. No sign of β-amyloid pathology, using immunohistochemical staining of SAMP8 hippocampi at the age of 6 months with rabbit anti-β-amyloid antibody (Invitrogen 71-5800). n = 4-5 mice per group, 8-10 sections per brain.

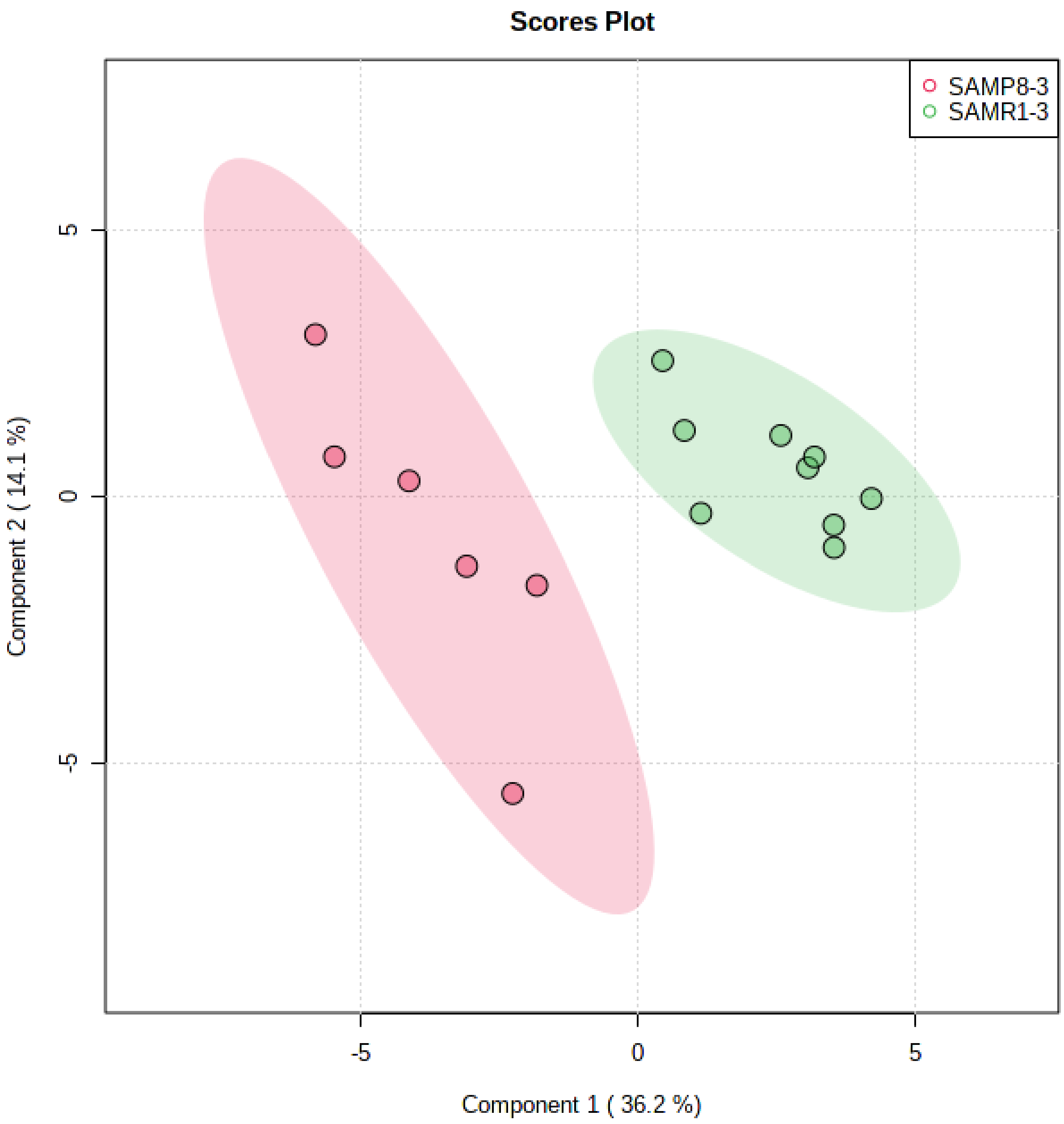

Supplementary Figure 3. PLS-DA model displayed group separation without any outlier; scores plot. The results of leave-one-out cross validation: number of components: 2, accuracy: 1.00, R2=0.93, Q2=0.82, p value of permutation test with 2000 repetitions = 0.106; color coding: SAMP8 (n = 6) in red, SAMR1 (n = 9) in green.
